# S05 Active ageing from theory to practice: success stories from Belgium, Finland, Germany and the Netherlands

**DOI:** 10.1093/eurpub/ckac093.022

**Published:** 2022-08-29

**Authors:** Katja Borodulin, Filip Boen, Liesbeth Preller, Heli Starck, Ute Mueller-Steck, Jannique van Uffelen

**Affiliations:** 1 Department of Physical Activity, Age Institute, Helsinki, Finland; 2 Department of Movement Sciences, KU Leuven, Leuven, Belgium; 3 Knowledge Centre for Sport & Physical Activity Netherlands, Utrecht, The Netherlands; 4 Department of Physical Activity, Age Institute, Helsinki, Finland; 5 Bildungsakademie des Landessportbundes Hessen, Frankfurt, Germany

**Keywords:** physical activity, older persons, implementation, volunteers, participation

## Abstract

**Introduction:**

Research increases our understanding on the efficacy of exercise and physical activity on older adults' health and well-being. However, the scientific output is relevant in experimental or clinical settings only and cannot be easily implemented in real-life setting. Consequently, the public health impact of the research output fails to be scaled-up for audiences and target groups at large. We therefore need more evidence on theory-to-practice approaches, in which physical activity interventions pay more attention to practical implementation.

**Aim:**

The aim of this symposium is to describe successful examples on active ageing from research-to-practice. Examples are from 4 countries where different approaches are being used to improve adoption, implementation and maintenance physical activity among older people. Relevant scientific frameworks like RE-AIM and implementation science are utilized.

